# Correctable biliary atresia and cholangiocarcinoma: a case report of a 63-year-old patient

**DOI:** 10.1186/s40792-019-0748-9

**Published:** 2019-11-29

**Authors:** Masaki Nio, Motoshi Wada, Hideyuki Sasaki, Hiromu Tanaka, Masatoshi Hashimoto, Yudai Nakajima

**Affiliations:** Department of Pediatric Surgery, Tohoku University Graduate School of Medicine, 1-1, Seiryo-machi, Aoba-ku, Sendai, 980-8574 Japan

**Keywords:** Biliary atresia, Correctable type, Long-term survivor, Cholangiocarcinoma

## Abstract

**Background:**

Although cancer occurrence following surgery for biliary atresia has gradually increased, the development of cholangiocarcinoma in a native liver survivor of biliary atresia is extremely rare.

**Case presentation:**

A 3-month-old female patient with the correctable type of biliary atresia underwent a cystoduodenostomy. At 16 years of age, she underwent multiple surgeries including lysis of intestinal adhesions, ileostomy, and gastrojejunostomy at another hospital. At 54 years of age, she underwent lithotomy at the porta hepatis, resection of the residual cystic bile duct with gallbladder, and hepaticojejunostomy in Roux-en-Y fashion. As she approached the age of 63, her computed tomography scan showed no liver tumors. In the following year, she developed cholangiocarcinoma at the porta hepatis and underwent chemotherapy. However, the cancer progressed, and she died before she reached the age of 64 years.

**Conclusions:**

Cholangiocarcinoma is extremely rare in patients with biliary atresia. However, physicians should follow up patients with biliary atresia as closely as possible, as malignant tumors secondary to biliary atresia may increase in number in the near future because of the growing number of long-term survivors with biliary atresia.

## Background

Correctable biliary atresia, for which hepaticoenterostomy is a feasible treatment, is associated with better surgical outcomes than the uncorrectable type, in which Kasai portoenterostomy is required [1]. However, the ultimate prognosis of biliary atresia remains uncertain in both correctable and uncorrectable biliary atresia. We recently treated a patient with native liver with correctable biliary atresia who developed cholangiocarcinoma and died at 63 years of age. She was the longest survivor of correctable biliary atresia in our series in the Tohoku University Hospital. Herein, we report her clinical course following surgery for biliary atresia and discuss the secondary malignancy arising in patients with biliary atresia.

## Case presentation

**Fig. 1:**
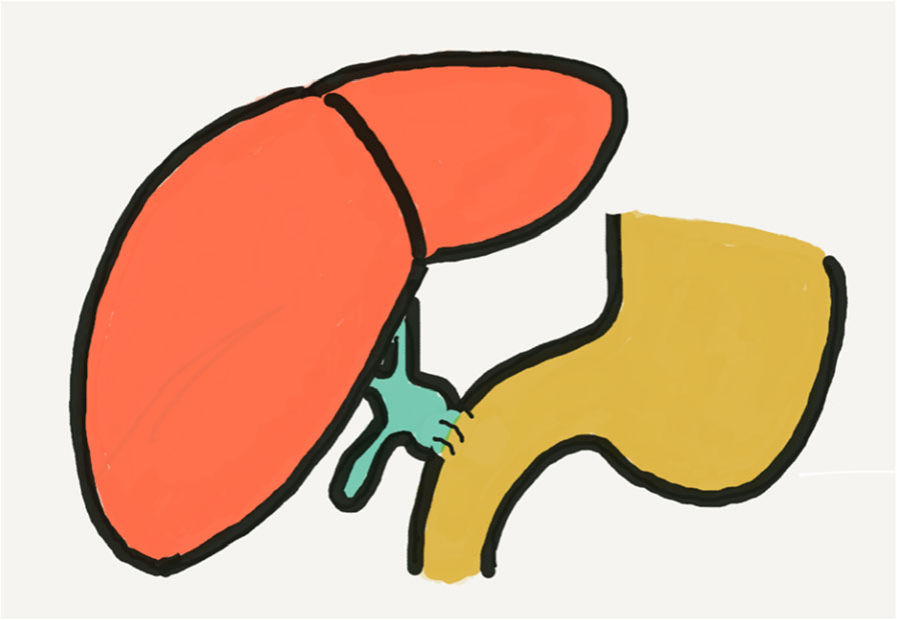
Cystoduodenostomy was performed for biliary atresia of a type I cyst at age 3 months. The gallbladder was not removed during the initial surgery

A 3-month-old girl with correctable biliary atresia with a cystic structure at the porta hepatis (Icyst-c1-α according to the Japanese Biliary Atresia Society Classification [2]) underwent cystoduodenostomy in the Tohoku University Hospital (Fig. 1). The jaundice she had experienced during the preoperative period disappeared following the surgery. She did not have health issues during childhood. At 15 years of age, she developed adhesion ileus and was admitted to a different hospital. In the following year, she underwent multiple surgeries including lysis of intestinal adhesion, ileostomy, closure of ileostomy, and gastrojejunostomy with Braun anastomosis in the same hospital. Diaphragmatic eventration was identified on her chest X-ray obtained in the hospital when she was aged 17 years.

**Fig. 2:**
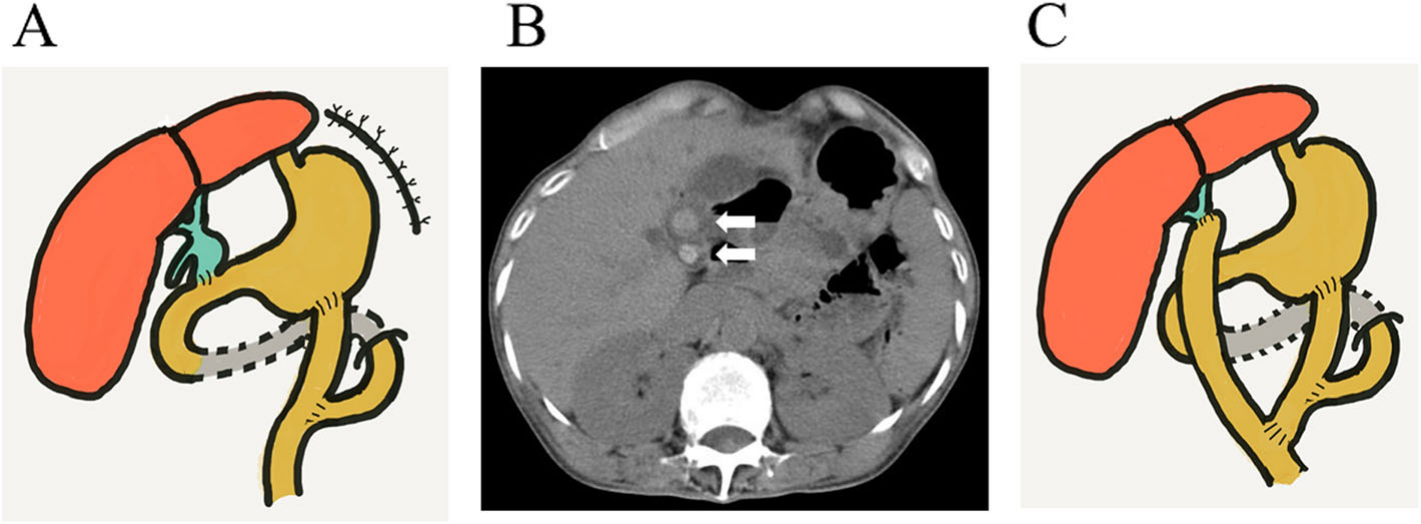
**a** The gastrojejunostomy was restored in a Roux-en-Y fashion at 31 years of age. Diaphragmatic plication was also performed. **b** Computed tomography scan shows gallstones at the porta hepatis at 54 years of age. White arrows: gallstones. **c** The cystic bile duct and gallbladder were resected. Additionally, biliary reconstruction was performed by hepaticojejunostomy in a Roux-en-Y fashion

At 31 years of age, she was referred to the Tohoku University Hospital because of poor general condition with ileus. Laboratory studies showed severe jaundice and malnutrition with total bilirubin level, 16.7 mg/dl; direct bilirubin level, 11.2 mg/dl; albumin level, 2.5 g/dl; and zinc level, 23 μg/dl. She developed generalized severe dermatitis due to zinc deficiency. She was diagnosed with volvulus affecting the jejunal loop where she underwent gastrojejunostomy; she subsequently developed blind loop syndrome. The dilated jejunal loop chronically pushed the left diaphragm upward, resulting in diaphragmatic eventration. The dilated jejunum was resected, and the gastrojejunostomy was restored in a Roux-en-Y fashion. Diaphragmatic plication was also performed (Fig. 2a). Thereafter, she was followed up in our department.

She had a habit of smoking 10 cigarettes/day for 34 years until 54 years of age, and then she quit smoking. She also had a habit of consuming a daily nightcap with small amount of alcohol. At 54 years of age, she developed cholangitis associated with gallstones at the porta hepatis (Fig. 2b), which were eventually removed. Following this, the residual cystic bile duct and gallbladder were resected, and biliary reconstruction was performed by hepaticojejunostomy in a Roux-en-Y fashion (Fig. 2c). No pathologically malignant or dysplastic changes were found in the resected specimens of the bile duct and gallbladder. We continued to follow up with the patient on a regular basis.

**Fig. 3:**
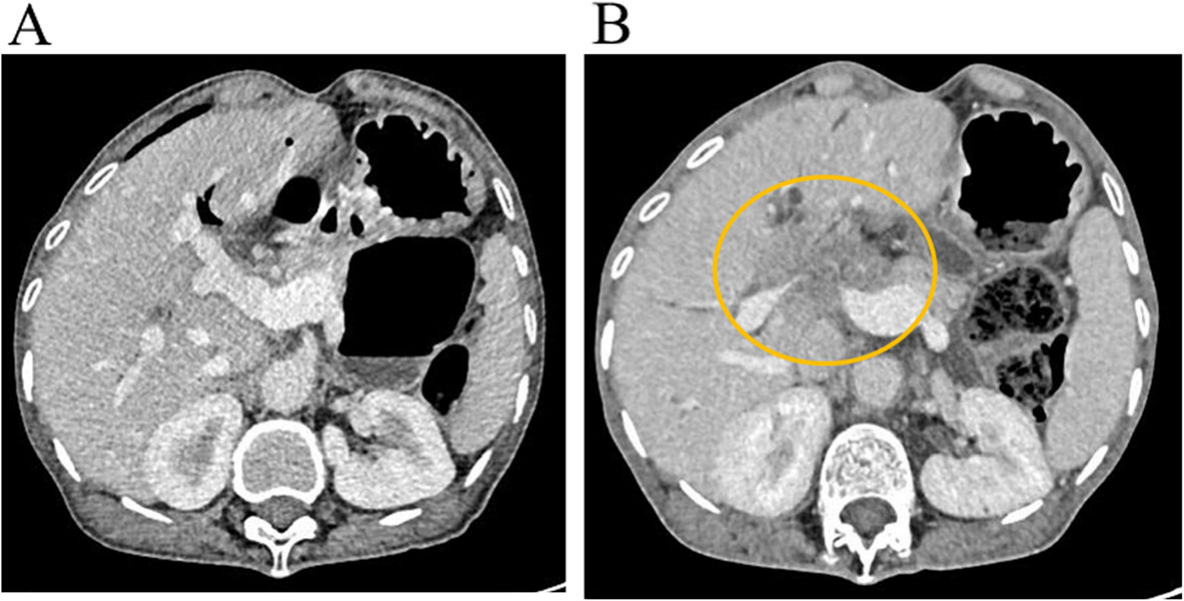
**a** Computed tomography was performed at a regular checkup. The scan shows no gallstones or tumor formation in the liver at 62 years and 10 months of age. **b** A liver tumor at the porta hepatis is revealed by computed tomography. The patient was aged 63 years and 6 months. Yellow circle: the tumor at the porta hepatis

At a follow-up assessment when the patient was approaching the age of 63, her liver function test results were within the normal range (Table 1). Computed tomography (CT) scan showed neither gallstones nor tumors in the liver (Fig. 3a).

**Table 1:** Laboratory study results

Patient age	62 years and 8 months	62 years and 10 months	63 years and 6 months
T.Bil^6)^ (mg/dl)	0.4	0.4	0.9
D.Bil^5)^ (mg/dl)	0.1	0.1	0.4
AST^3)^ (IU/l)	37	12	56
ALT^2)^ (IU/l)	28	28	67
AFP^1)^ (ng/ml)	2.6	No data	1.8
CEA^4)^ (ng/ml)	4.8	No data	7
CA19-9 (U/ml)	15.8	No data	301.2

Her liver function tests were within the normal range at 62 years and 8 months old and 62 years and 10 months old. Her liver function test results showed slight abnormalities at 63 years and 6 months old. Among tumor markers assessed at that time, CEA and CA19-9 levels were elevated; both markers were within normal range 10 months before

*AFP*^*1)*^ α-fetoprotein, *ALT*^*2)*^ alanine aminotransferase, *AST*^*3)*^ aspartate aminotransferase, *CEA*^*4)*^ carcinoembryonic antigen, *D. Bil*^*5)*^ direct bilirubin, *T. Bil*^*6)*^ total bilirubin

**Fig. 4:**
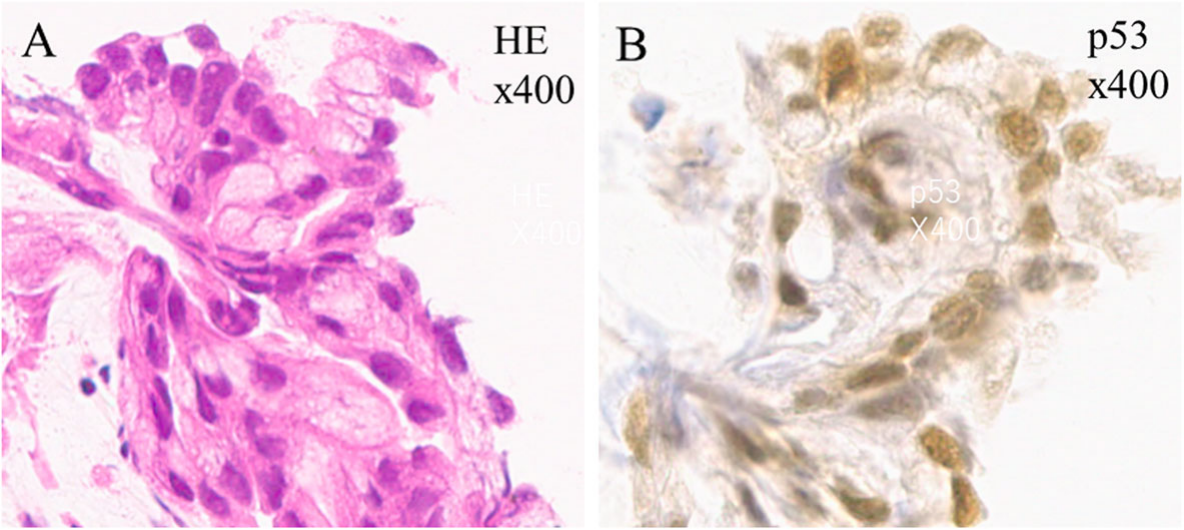
**a** Hematoxylin-eosin (HE) staining. Pathological assessment of the tumor specimens obtained by endoscopic biopsy reveal adenocarcinoma (× 400). **b** p53 immunohistochemical staining. The tumor cells are positive for p53 staining (× 400)

However, at 8 months later, her liver function test results showed slight abnormalities (Table 1). Among tumor markers assessed at that time, carcinoembryonic antigen and cancer antigen 19-9 levels were elevated; both markers were within normal range 10 months before (Table 1). Ultrasonography showed dilated intrahepatic bile ducts, and a liver tumor at the porta hepatis was found by CT (Fig. 3b). Pathological specimens obtained by endoscopic tumor biopsy showed adenocarcinoma (Fig. 4a, b). She was diagnosed with cholangiocarcinoma that was already progressive and unresectable. Although she underwent cancer chemotherapy with gemcitabine, the tumor rapidly progressed. She died just before the age of 64 years. The postmortem autopsy was not performed, according to the will of her family.

## Conclusions

Clinical outcomes of correctable biliary atresia are believed to be better than those of the uncorrectable type [1]. However, few reports have been published regarding long-term results of correctable biliary atresia [3, 4], and the ultimate prognosis is still uncertain. In the current report, we presented a 63-year-old female patient with correctable biliary atresia. To the best of our knowledge, she was the world’s longest survivor of correctable biliary atresia with a native liver. She underwent the initial surgery several years before the first Kasai portoenterostomy for biliary atresia was performed in our department in 1957 [5]. Currently, standardized surgery for the cystic type of biliary atresia is hepaticoenterostomy or Kasai portoenterostomy after resection of the cystic bile duct. She underwent cystoduodenostomy that preserved the gallbladder, which is an unusual procedure from the current standpoint. In the mid-1950s, the common procedure for choledochal cyst was not the removal of the cystic bile duct, but internal biliary drainage by cystoduodenostomy. Thus, the use of that procedure for this patient cannot be criticized. However, when she underwent a laparotomy at 31 years of age, total removal of the cystic bile duct could have been considered. As it was practically difficult because of her severely affected general condition, only resection of the dilated jejunal loop and diaphragmatic plication were performed.

Cancer occurrence following surgery for biliary atresia has gradually increased. Yoon et al. reviewed a total of 32 malignancies arising in patients with biliary atresia [6]. Among them, 25 were hepatocellular carcinoma secondary to liver cirrhosis that developed in survivors with a native liver or were found in the removed liver from patients after liver transplantation.

In the remaining seven cases, five had cholangiocarcinoma and two had hepatoblastoma. Until now, seven cases of cholangiocarcinoma in patients with biliary atresia have been reported, including the present case [6–11] (Table 2).

**Table 2:** Cholangiocarcinoma in biliary atresia patients

Case (reference number, publication year)	Age at diagnosis (years)	Sex	Treatment	Length of survival following diagnosis (months)	Outcome
1 ([7], 1968)	3	M	-	(Autopsy)	Dead
2 ([8], 1977)	11	F	-	(Autopsy)	Dead
3 ([9], 2012)	16	F	LT^2)^/Chemo^1)^	33	Alive (with Meta^3)^)
4 ([10], 2013)	30	F	LT^2)^/Chemo^1)^	7	Dead
5 ([6],2014)	13	F	LT^2)^/Chemo^1)^	11	Dead
6 ([11], 2016)	39	M	Chemo^1)^	8	Dead
7 Present case	62	F	Chemo^1)^	4	Dead

Seven cases of cholangiocarcinoma in patients with biliary atresia have been reported, including the present case

*Chemo*^*1)*^ chemotherapy, *LT*^2)^ liver transplantation, *Meta*^*3)*^ metastasis

This includes two previous cases reported in 1968 and 1977 in which the corrective surgery for biliary atresia was not performed (Table 2, cases 1 and 2). In two other cases, cancers were coincidentally detected in removed livers from recipients during liver transplantation (Table 2, cases 3 and 4). Another patient developed cholangiocarcinoma and underwent liver transplantation (Table 2, case 5). The remaining two survivors with a native liver, including the present case, developed cholangiocarcinoma during the long-term follow-up after corrective surgery for biliary atresia (Table 2, cases 6 and 7). In case 6, a 39-year-old male patient developed cholangiocarcinoma simultaneously combined with hepatocellular carcinoma.

Six patients died within a year after the diagnosis of cholangiocarcinoma. The remaining patient, in whom cholangiocarcinoma was coincidentally detected in the removed liver after liver transplantation, was alive and had multiple metastases at the time of publication (Table 2, case 3).

In the surgical treatment of a choledochal cyst, both hepaticojejunostomy and hepaticoduodenostomy were commonly employed. However, some authors advocated that hepaticoduodenostomy should not be used because of the higher risk of postoperative complications including reflux gastritis, cholangitis, and carcinogenesis [12–14]. Others supported hepaticoduodenostomy because no evidence of a higher incidence of complications following hepaticoduodenostomy had been demonstrated yet [15]. In the present case, the remaining cyst that was anastomosed with the duodenum at the initial surgery was removed at 54 years of age. The removed cyst and gallbladder were pathologically assessed, and no malignant or dysplastic changes were revealed at that time. We believe that hepaticojejunostomy should be employed for correctable biliary atresia rather than hepaticoduodenostomy to prevent long-term morbidities including cholangitis and hepatolithiasis, which are relatively common late complications, even in type I biliary atresia [16]. Although there was no evidence of carcinogenesis directly due to cystoduodenostomy in this case, repeated cholangitis and gallstone formation at the porta hepatis, which might have been associated with the residual cyst, were possible cancer-inducing factors. Thus, it might have been better to remove the cyst and switch the drainage route from cystoduodenostomy to hepaticojejunostomy in a Roux-en-Y fashion for the present patient in the earlier stage.

Smoking and drinking were reported as risk factors of intrahepatic cholangiocarcinoma [17]. As the current patient had habits of smoking and drinking, these factors might have had an effect on carcinogenesis.

Regarding the follow-up protocol of non-symptomatic adult patients, they were advised to visit our outpatient clinic every 6 months or annually. We routinely evaluate blood chemistry parameters including tumor markers. Ultrasonography is also used during every visit. If the patient has any symptoms or signs, further diagnostic modalities, such as CT and magnetic resonance imaging, are performed.

Despite the close follow-up with regular checkups, early diagnosis of cholangiocarcinoma was not possible in the present case. Although regular checkups of tumor markers within shorter intervals might have made an earlier diagnosis of cholangiocarcinoma possible, it would be difficult to justify the inclusion of such frequent assessment of tumor markers in a follow-up regimen of patients with biliary atresia in clinical practice.

Cholangiocarcinoma is extremely rare in patients with biliary atresia. However, physicians should keep doing their best to follow up with patients with biliary atresia as closely as possible, as malignant tumors secondary to biliary atresia may increase in number in the near future because of the growing number of long-term survivors with biliary atresia.

## Data Availability

This manuscript is a case report and the personal data of the electric chart will not be shared because no permission was obtained from the ethics committee.

## Abbreviations

AFP: α-Fetoprotein
ALT: Alanine aminotransferase
AST: Aspartate aminotransferase
CA: Cancer antigen
CEA: Carcinoembryonic antigen
CT: Computed tomography
D.Bil: Direct bilirubin
T.Bil: Total bilirubin
